# Identification of Co-Expressed Genes Related to Theacrine Synthesis in Tea Flowers at Different Developmental Stages

**DOI:** 10.3390/ijms222413394

**Published:** 2021-12-13

**Authors:** Xiaomin Chen, Shuxian Shao, Ruxing Yang, Mengya Gu, Pengjie Wang, Feng Zhao, Naixing Ye

**Affiliations:** 1 College of Horticulture, Fujian Agriculture and Forestry University, Fuzhou 350002, China; 1190311002@fafu.edu.cn (X.C.); 1200311010@fafu.edu.cn (S.S.); 1190311005@fafu.edu.cn (M.G.); 2180311002@fafu.edu.cn (P.W.); 2 Tea Research Institute, Fujian Academy of Agricultural Sciences, Fuzhou 350012, China; yangrx@faas.cn; 3 Genome Analysis Laboratory of the Ministry of Agriculture, Agricultural Genomics Institute at Shenzhen, Chinese Academy of Agricultural Sciences, Shenzhen 518120, China; 4 College of Pharmacy, Fujian University of Traditional Chinese Medicine, Fuzhou 350122, China

**Keywords:** tea flower, purine alkaloid pathway, theacrine, transcriptomic, WGCNA

## Abstract

Jiaocheng kucha is the first reported tea germplasm resource which contains theacrine founded in Fujian Province. Currently, the anabolic mechanism of theacrine within tea leaves is clear, but there are few studies focused on its flowers. In order to further explore the mechanism of theacrine synthesis and related genes in flowers, current study applied Jiaocheng kucha flowers (JC) as test materials and Fuding Dabaicha flowers (FD) as control materials to make transcriptome sequencing, and determination of purine alkaloid content in three different developmental periods (flower bud stage, whitening stage and full opening stage). The results showed that the flower in all stages of JC contained theacrine. The theacrine in the flower bud stage was significantly higher than in the other stages. The differentially expressed genes (DEGs) at three different developmental stages were screened from the transcriptome data, and were in a total of 5642, 8640 and 8465. These DEGs related to the synthesis of theacrine were primarily annotated to the pathways of purine alkaloids. Among them, the number of DEGs in xanthine synthesis pathway was the largest and upregulated in JC, while it was the smallest in caffeine synthesis pathway and downregulated in JC. Further weighted gene co-expression network (WGCNA) indicated that *ADSL* (*CsTGY03G0002327), ADSL* (*CsTGY09G0001824) and UAZ* (*CsTGY06G0002694*) may be a hub gene for the regulation of theacrine metabolism in JC. Our results will contribute to the identification of candidate genes related to the synthesis of theacrine in tea flowers, and explore the molecular mechanism of theacrine synthesis in JC at different developmental stages.

## 1. Introduction

Bitter tea is a kind of unique plant which mainly distributed in Yunnan, Hunan, Guizhou, Fujian and some other provinces of China [1]. It is quite different from existing tea resources due to its ultra-bitter taste. Currently, there are several known reasons that could be attributed to such bitterness, such as abundance in purine alkaloids, flavonoids, anthocyanins, or saponins [2]. It is worth noting that one class of bitter tea which contains theacrine has attracted extensive attention. Theacrine (1,3,7,9-tetramethyluric acid) is an alkaloid compound accompany with bioactive functions such as anti-depression, sedation, hypnosis, lipid metabolism regulation, etc. [3]. It is also the second richest purine alkaloid in bitter tea [4]. As caffeine could convert to theacrine by oxidation at C8 and methylation at N9 in certain tea germplasm resource [5], theacrine is also regarded as a downstream metabolite of caffeine. As the formation pathway and related regulatory genes of theacrine have been studied well [6,7,8,9,10,11], N-methyltransferase encoded by the gene *TEA024443* could be the key enzyme for synthesis of theacrine [12]. Meanwhile, 9-N-methyltransferase (*CKTCS*) was identified as being involved in synthesis process of theacrine by transcriptome sequencing of N-methyltransferase in Pu’er (*Camellia sinensis* var. *assamica*) and kucha [13].

Jiaocheng kucha is the first reported tea with abundant theacrine, found in the Fujian Province. In our previous work, transcriptome was applied in studying Jiaocheng kucha’s leaves. KEGG pathway analysis annotated differential expression genes to four metabolic pathways, including guanine degradation, adenine degradation, caffeine synthesis and caffeine degradation. Further analysis indicated that urate oxidase *UOX (TEA012458)* is downregulated, while tea caffeine synthase *TCS* (*TEA030024*) is upregulated in Jiaochengkucha [14].

Commonly, the buds and tender leaves of *Camellia sinensis* are the most valuable part, whereas few studies focus on its flowers [15]. Tea flowers contain a variety of substances beneficial to the human body and are becoming more and more widely used, such as extraction of tea flower, utilization of tea pollen products and tea flower food processing [16,17]. Specifically, Zhao et al. [18] used tea flowers as raw materials to prepare iced tea, which could be refreshing with a marvelous fragrance. Zhang et al. [19] developed tea flower soft candy which is fragrant and elegant. Previous studies mainly focused on biochemical components and physiology-related genes of tea flowers. It is also found that tea flowers could contain less catechin and caffeine than tea leaves [20,21]. Previously, genes related to the synthesis of catechins, amino acids and anthocyanins in tea flowers have been studied [22,23,24]; however, the purine alkaloid metabolism of tea flowers is yet to be elucidated. Here, we applied Jiaocheng kucha flowers (JC) as test materials and Fuding Dabaicha flowers (FD) as control materials to explore the synthesis mechanism and related regulatory genes. Transcriptome sequencing and determination of purine alkaloid content were performed throughout a total of three developmental periods (flower bud stage, whitening stage and full opening stage).

## 2. Results

### 2.1. Quantification of Purine Alkaloids in Different Flowering Periods between JC and FD

**Figure 1:**
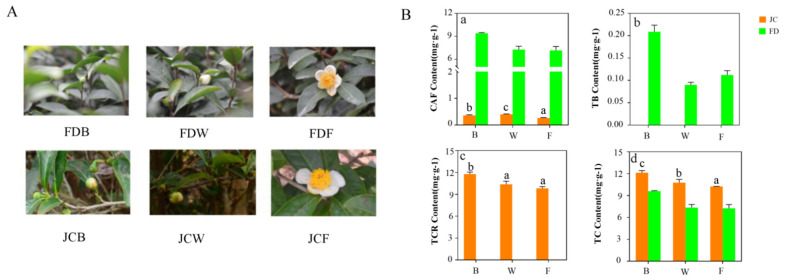
Morphological characteristics of tea blossom (**A**). JCB, FDB is the flower bud stage. JCW, FDW is the whitening stage; JCF, FDF is the full opening stage. Purine alkaloids content in Kucha (JC) and conventional varieties (FD); (**B**). a. caffeine content (CAF); b. theobromine content (TB); c. theacrine content (TCR); d. the total content of purine alkaloids (TC). ‘B’ is the flower bud stage, ‘W’ is the whitening stage, ‘F’ is the full opening stage.

Figure 1A defined different flowering periods of JC and FD. Related purine alkaloid contents were shown in Figure 1B. The caffeine contents of JC in the three stages were 0.36, 0.41 and 0.26 mg/g, respectively, which were lower than that of FD. There were significant differences in its caffeine contents. The highest content was found in the whitening stage, and the lowest was found in the full opening stage. Theobromine was not detected in JC at all. Interestingly, theacrine was detected at all flowering stages of JC, and the content was 11.78, 10.37 and 9.83 mg/g, respectively. The flower bud stage contained the highest theacrine. For the total amount of purine alkaloids, JC was higher than FD in all three stages.

### 2.2. Transcriptome Data Analysis

A total of 18 libraries were constructed (Table 1), and 143.41 Gb of clean reads were constructed with an average value of 7.97 Gb in each sample. All testing samples Q20 value were over 98.45%, and Q30 value were over 95.19%. The GC average content values were 45.14%, and those uniquely mapped were 83.62%, respectively.

**Figure 2:**
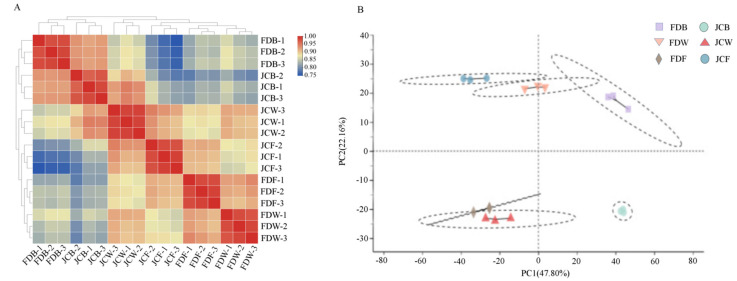
Pair-wise Pearson correlation coefficients of the sequencing data from three replicates × two genotypes in three periods (**A**). Principal component analysis (PCA) of transcriptome data (**B**).

Pearson analysis (Figure 2A) indicated that there was high repeatability. PCA clustered samples into six groups. FD and JC were clearly distinguished. Meanwhile, different flowering periods were also distinguished well (Figure 2B).

### 2.3. Identification of Differentially Expressed Genes (DEGs)

**Figure 3:**
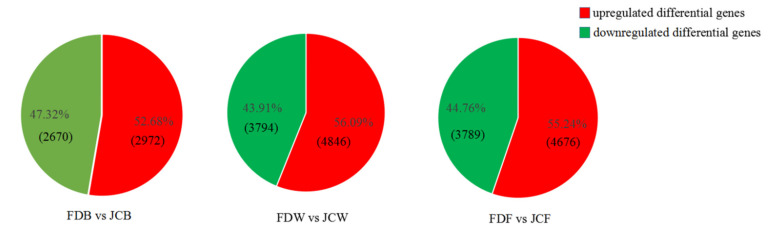
DEGs in each period. Red is upregulated DEGs and green is downregulated DEGs. The number of DEGs is in parentheses.

As shown in Figure 3, FDB vs. JCB had 5642 DEGs (2972 upregulated and 2670 downregulated), FDW vs. JCW had 8640 DEGs (4846 upregulated and 3794 downregulated), FDF vs. JCF had 8465 DEGs (4676 upregulated and 3789 downregulated). The proportion of upregulated DEGs in all three periods were 52.68, 56.09 and 55.24% respectively, while the proportion of downregulated DEGs in all three periods were 47.32, 43.91 and 44.76%, respectively. The number of upregulated DEGs was higher than downregulated DEGs in all comparisons. Interestingly, the number of DEGs in FDB vs. JCB was far less than other comparisons, with the same number of DEGs in FDW vs. JCW and FDF vs. JCF (Figure 3).

### 2.4. GO and KEGG Enrichment Analysis of DEGs

**Figure 4:**
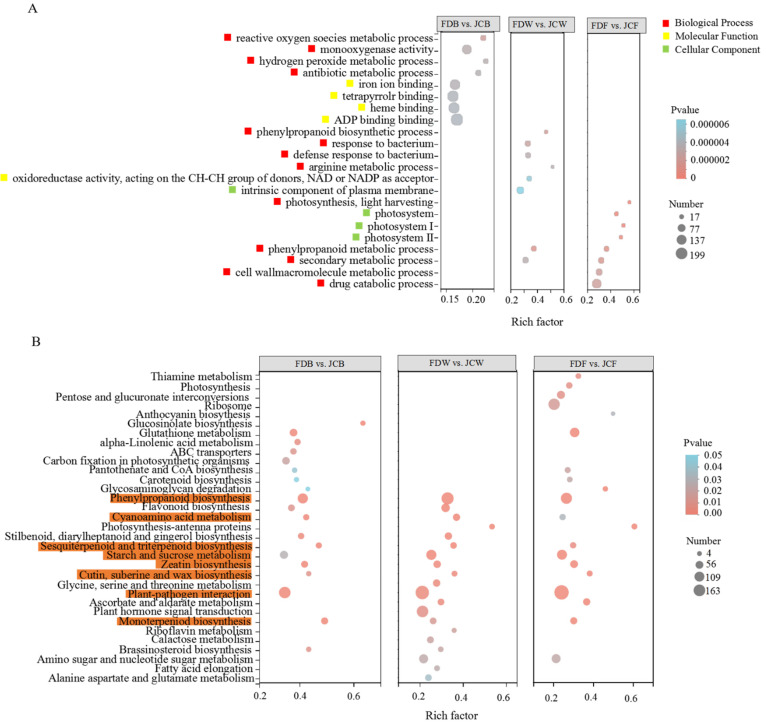
Go enrichment analysis of DEGs (**A**) and KEGG enrichment analysis of DEGs (**B**). Note: The metabolic pathways shown in red are common to all three periods.

There were significant differences in GO enrichment among all three stages (Figure 4A). DEGs at the flower bud stage were mainly concentrated in BP and MF, and the dominant terms were monooxygenase activity, iron ion binding, tetrapyrrole binding, heme binding and ADP binding. In whitening stage, DEGs were mainly concentrated in MF, and the dominant terms were response to bacteria, defense response to bacteria, phenylpropanoid metabolic process and secondary metabolic process. Interestingly, in CC (intrinsic component of the plasma membrane), more differentially expressed genes were also enriched. The full opening period was mainly concentrated in CC and BP, and the dominant terms were drug catabolic process, cell wall macromolecule metabolic process and photosystem.

125, 130, and 131 metabolic pathways identified by KEGG enrichment analysis in the FDB vs JCB, FDW vs JCW, and FDF vs JCF. Eight metabolic pathways of standard red in Figure 4B were enriched in three phases simultaneously. Among them, phenylpropanoid biosynthesis and plant-pathogen interactions were more enriched in DEGs.

**Figure 5:**
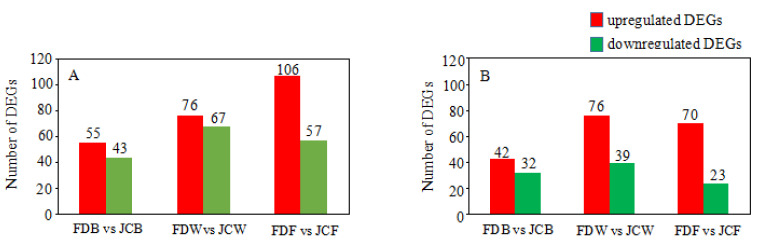
The number of DEGs in plant–pathogen interaction (**A**) and phenylpropanoid biosynthesis (**B**).

Further analysis of pathways with the largest number of DEGs was performed. In plant–pathogen interaction, there were 98, 143 and 163 DEGs enriched, respectively. In phenylpropanoid biosynthesis, there were 74, 115 and 93 DEGs enriched, respectively. In both pathways, there were more upregulated genes than downregulated genes (Figure 5).

### 2.5. DEGs Analysis in the Purine Alkaloid Pathway

**Figure 6:**
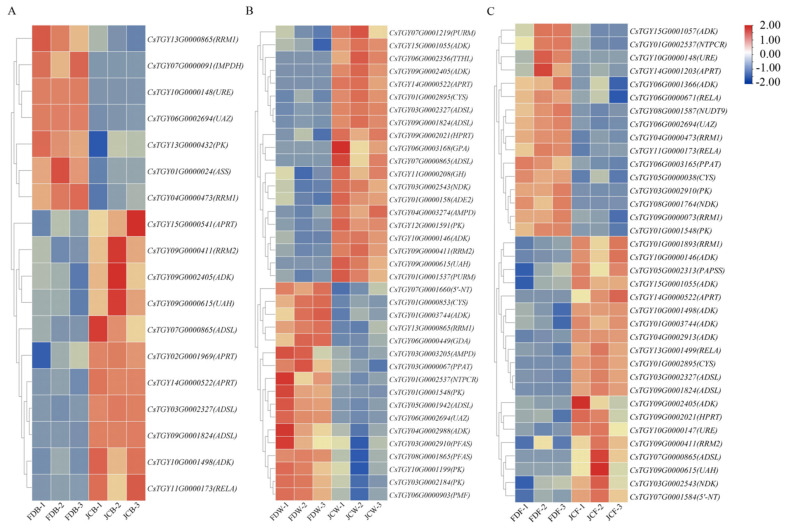
The heatmap of DEGs in the synthesis pathway of purine alkaloids. DEGs in flower bud stage (**A**), DEGs in whitening stage (**B**), DEGs in full opening stage (**C**).

For tea plants, the most common existing purine alkaloid is caffeine, with a small amount of theobromine and trace theophylline. Theacrine is usually existent in certain unique bitter tea plants. The synthesis pathway of purine alkaloids has an important influence. Xanthine could convert to 7-methylxanthine by N-methyltransferase, which in turn forms theobromine by *TCS*. Theobromine could convert to caffeine by *TCS*. The downstream metabolite of caffeine in common tea varieties is theophylline, while theacrine is also one of the metabolites of caffeine in bitter tea varieties. Figure 6 indicated genes related to purine alkaloid synthesis. The number of genes enrichment in purine alkaloid pathway was different throughout the flowering stages. It was obvious that most DEGs were related to xanthine synthesis, while there were few DEGs associated with caffeine synthesis and metabolism. There were 18, 37 and 36 DEGs at the flower bud stage, whitening stage and full opening stage. DEGs were mainly Adenosine kinase (*ADK*), Adenylosuccinate lyase (*ADSL*), etc. The expression of these genes was varied in different flowering stages and cultivars.

**Figure 7:**
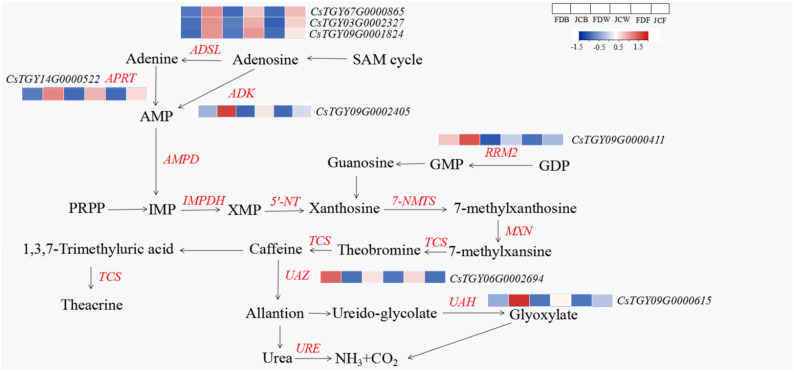
Heatmap of common DEGs in three stages.

Three flowering stages had eight common DEGs, including one *ADK* (*CsTGY09G0002405*), three *ADSL* (*CsTGY07G0000865*, *CsTGY03G0002327*, *CsTGY09G0001824*), one *RRM2* (*CsTGY09G0000411*), one *APRT* (*CsTGY14G0000522*), one *UAZ* (*CsTGY06G0002694*) and one *UAH* (*CsTGY09G0000615*), which were differential in different genotypes and different stages (Figure 7). There were six genes belonging to the xanthine pathway, and the expression levels of genes at three periods were all upregulated in JC. Interestingly, the expression of these genes showed a downward trend at different flowering stages, with the peak achievement at flower bud stage, while the lowest point was at the full opening stage. This was consistent with the accumulation trend of theacrine in JC. The other two genes were related to caffeine metabolism pathway. *UAZ* (*CsTGY06G0002694*) was urate oxidase that promotes the degradation of caffeine into allantion. The expression of *UAZ* in JC were lower than that in FD in all periods, which was similar to caffeine accumulation during tea plant blossoms. *UAH* (*CsTGY09G0000615*) was ureidoglycolate hydrolase that promotes the degradation of ureido-glycolate into glyoxylate. The expression of *UAH* in JC was higher than that in FD during flowering.

### 2.6. Co-Expression Network Analysis

**Figure 8:**
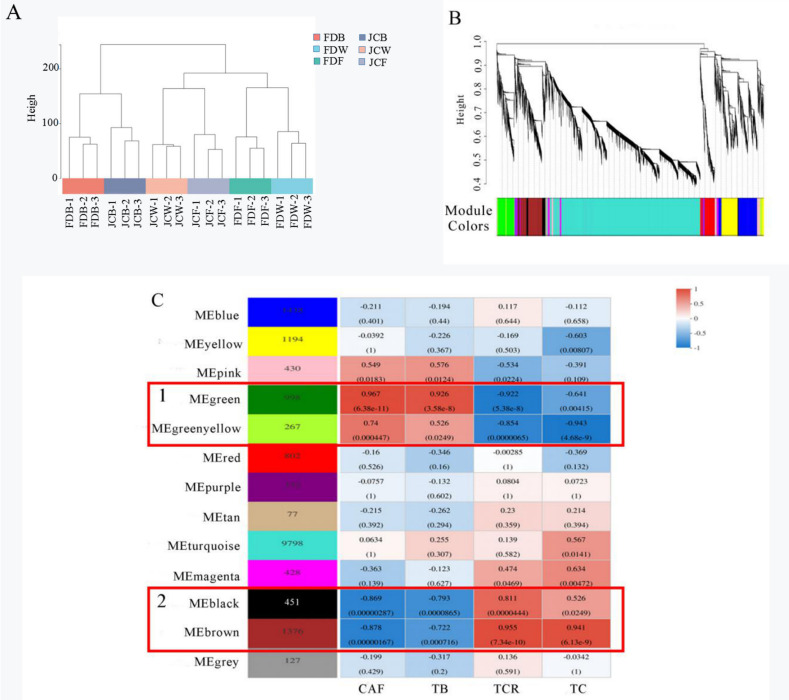
Co-expression network analysis. (**A**) is the sample clustering tree; (**B**) is the gene clustering tree and module cutting. Each branch of the gene clustering tree corresponds to a module. (**C**) is correlation analysis between modules and phenotypes, box 1 of two modules (green and green–yellow) were significantly positively correlated with caffeine and theobromine contents; box 2 of two modules (black and brown) were significantly positively correlated with theacrine and total amount of purine alkaloids.

After filtering genes with TPM < 17, a total of 718 differential genes, a weighted gene co-expression network was constructed. The expression levels of 18 samples were calculated and performed with clustering analysis. As shown in Figure 8A, clustering among the samples was good without outlier samples. Given that the scale-free tolerance curve is smooth, the power exponential weighted β value—the soft threshold—was set as 12.

Correlation analysis and clustering were performed according to the TPM values of the different genes. Then the genes with higher correlation were assigned to a module. As shown in Figure 8, all genes were divided into 13 modules. The gray color represents genes that were not divided into other modules (Figure 8B).

Figure 8C showed the number of genes contained in each module and the corresponding correlation with purine alkaloids content. The number of genes contained in different modules varied greatly; the turquoise module had the largest number of genes (9798), while the tan module had the lowest number (77). The modules with a large correlation coefficient (absolute value) and small significant *p* value were significantly correlated with phenotypes. The compounds identified as highly significantly associated with purine alkaloids were marked green, green–yellow (Box 1), black, and brown (Box 2). Interestingly, green and green–yellow were significantly positively correlated with both caffeine and theobromine contents, while theacrine and the total amount of purine alkaloids were significantly negatively correlated. Black and brown were significantly positively correlated to both theacrine and the amount of purine alkaloids but negatively correlated to caffeine and theobromine.

KEGG analysis was performed on genes of four modules, and the top 20 metabolic pathways with the highest enrichment degrees were screened out, as shown in the Supplementary Tables (Table S1). The genes of these four modules were mainly enriched in carbohydrate metabolisms, which were glycolysis, gluconeogenesis and starch and sucrose metabolism. The rest of the genes were related to environmental adaptation, folding, sorting and degradation and signal transduction. Notably, nucleotide metabolism was enriched in both green and brown modules, which mainly corresponded to pyrimidine and purine metabolism. Purine metabolism was the main metabolic pathway of caffeine, theobromine, and theacrine synthesis. Related genes in these pathways may have a certain relationship with accumulation of them.

**Figure 9:**
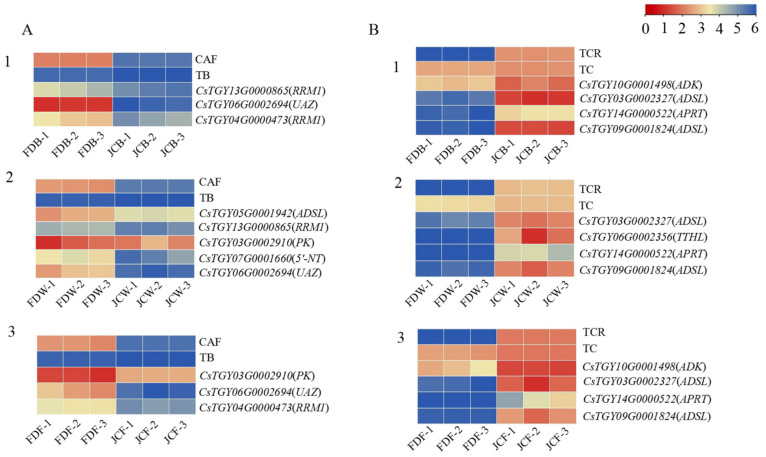
Heatmap of DEGs of purine alkaloid pathway in green module and the content of CAF and TB (**A**). Heatmap of DEGs of purine alkaloid pathway in brown module and the content of TCR and TC (**B**). 1. DEGs in flower bud stage; 2. DEGs in whitening stage; 3. DEGs in full opening stage.

A total of six DEGs were enriched in the green module of purine alkaloid pathway. Six DEGs in the green module were *ADSL* (*CsTGY05G0001942*), *RRM1* (*CsTGY13G0000865*), *PK* (*CsTGY03G0002910*), *5′-NT* (*CsTGY07G0001660*), *UAZ* (*CsTGY06G0002694*) and *RRM1* (*CsTGY04G000473*). Their expression quantities were all decreased in JC. They were also expressed inconsistently at the three flowering stages. At the flower bud stage, there were only three DEGs: *RRM1* (*CsTGY13G0000865*), *UAZ* (*CsTGY06G0002694*) and *RRM1* (*CsTGY04G000473*). There were five DEGs (*ADSL*, *RRM1*, *PK*, *5′-NT* and *UAZ*) in the whitening stage. There were only three DEGs found at the full opening stage, which were *PK* (*CsTGY03G0002910*), *UAZ* (*CsTGY06G0002694*) and *RRM1* (*CsTGY04G000473*). The green module was significantly correlated to caffeine and theobromine. During all flowering stages, the purine alkaloids enriched by this module were downregulated in JC, and the contents of caffeine and theobromine in JC were also significantly lower than in FD. The expression of these genes was positively correlated to accumulation of both caffeine and theobromine (Figure 9A).

Five DEGs in the brown module were related to purine alkaloid pathway, which includes *ADK* (*CsTGY10G0001498*), *ADSL* (*CsTGY03G0002327*), *TTHL* (*CSTGY06G0002356*), *APRT* (*CsTGY14G0000522*) and *ADSL* (*CsTGY09G0001824*). Their expression was upregulated in JC accompanied with all flowering stages. Except for *TTHL* (*CsTGY06G0002356*), the other four genes were expressed differentially between the flower bud and flowering stages. At the whitening stage, only *ADK* (*CsTGY10G0001498*) showed no differential expression. The brown module was significantly correlated to both theacrine and total amount of purine alkaloids. During all flowering stages, the purine alkaloids enriched by this module were upregulated in JC; furthermore, the contents of theacrine and total amount of purine alkaloids were also significantly higher than FD. The expression of these genes was positively correlated with the accumulation of theacrine and purine alkaloids content (Figure 9B).

### 2.7. Analysis of DEG Expression Level by PCR

**Figure 10:**
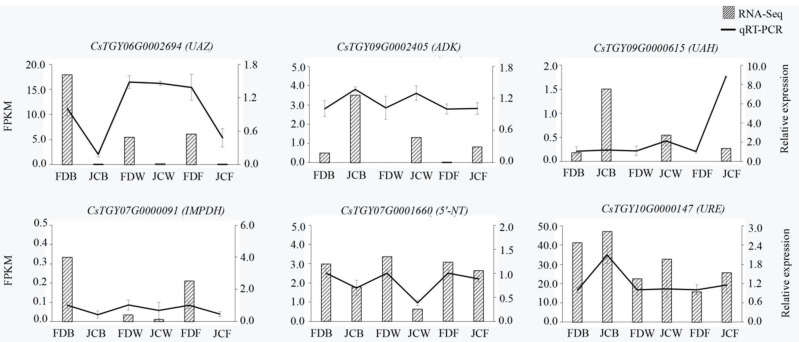
Validation of transcriptome sequencing data by qRT-PCR.

To further validate the reliability of transcriptome sequencing results, six genes were screened and identified, two of which were common to each of the three stages (*ADK*, *UAH*); one gene was common to all three stages in green (*UAZ*) and the other three genes were specific to all stages (*IMPDH, 5′-NT, URE*). Table S2 gave the result of primers for qRT-PCR. As shown in Figure 10, the relative expression of six DEGs was consistent with the trend of transcriptome sequencing, indicating that the transcriptome sequencing results could be reliable.

## 3. Discussion

Bitter tea is a kind of unique tea plant, due to its extremely bitter taste. Previous related research on this plant was mainly focused on its leaves, while little research has been carried out on its flowers. In our previous study, the purine alkaloids in Jiaocheng kucha leaves were quantified. In current study, the purine alkaloid components of its flowers were also quantified. As a result, its purine alkaloids were significantly higher (*p* < 0.05) than that of FD at all flowering stages, while the caffeine content was lower. Theacrine was detected in all the flowering stages of JC, with its peak level in the young bud stage (*p* < 0.05). In different flowering periods, the total amount of theacrine and purine alkaloids showed a downward trend, which is consistent with Lin et al. [25].

Tea plant genomes of different species were published [26,27,28]. Meanwhile, Zheng et al. demonstrated the synthetic pathway of theacrine [5]. Li and Wang [9,12] used bitter tea resources found in Jiangxi and Guangdong to perform transcriptome analysis, aiming at exploring the theacrine formation mechanism. Our group also conducted transcriptome to screen such related genes [14], as tea flowers of Jiaocheng kucha applied to our current research. The GO enrichment results were consistent with previous studies, confirming DEGs were mainly enriched in the biological process. During the flower bud stage, a large number of differential genes appeared in the Molecular Function among all flowering stages. The KEGG enrichment results showed that DEGs of tea flowers in JC were mainly enriched in phenylpropanoid biosynthesis, cyanoamino acid metabolism, sesquiterpenoid and plant–pathogen interaction. Especially in both the whitening and full opening stage, there were several DEGs related to plant–pathogen interaction, and most of the expressions in JC were upregulated. It was concluded that the resistance of JC may be higher than FD. Wang et al. found that N-methyltransferase (*NMT*) encoded by *TEA024443* may catalyze the methylation at 9-N position in bitter tea [12]. Li et al. speculated that two genes (*TEA010054* and *TEA022559*) might catalyze the final methylation step during the theacrine synthesis of bitter tea [9]. Zhang et al. identified the theacrine synthase *CKTCS* from bitter tea [13]. Previous studies revealed that N9-methyltransferase plays an essential role in the synthesis of theacrine. *TCS* and *NMT* were the key regulatory genes, most of which were related to caffeine anabolic pathway, whereas in our study, there were few differentially expressed genes in the caffeine metabolism pathway. The number of DEGs was different at different flowering periods; furthermore, the number of DEGs was the highest at the whitening stage (37). These DEGs were mainly *ADK*, *5’-NT*, *AMPD*, etc., which were upregulated in JC. For bitter tea leaves, key genes related to theacrine synthesis were commonly concentrated in caffeine synthesis or metabolic pathways, such as *NMT* and *TCS*. For bitter tea flowers, the genes related to its synthesis were mainly concentrated in the xanthine synthesis pathway, which is also the upstream pathway of caffeine synthesis, such as *ADSL*, *APRT*, *ADK* and *RRM2*. However, there were few differentially expressed genes in the caffeine biosynthesis pathway; *NMT* and *TCS* were not differentially expressed. Only three DEGs existed in the caffeine metabolism process; they were *UAZ* (*CsTGY06G0002694*), *UAH* (*CsTGY09G0000615*) and *URE* (*CsTGY10G0000148*), but the effect of these genes on theacrine synthesis was unclear. In conclusion, the genes related to the regulation of theacrine accumulation were different from leaves to flowers, although they all belong to a same purine alkaloid pathway.

Using WGCNA analysis, genes associated with target traits can be specifically screened and modularly classified to obtain co-expression modules of high biological significance [29]. In this study, the transcriptome was correlated with purine alkaloid component content data using the WGCNA method and the modules were subjected to KEGG functional enrichment analysis, resulting in the screening of four key modules that were highly correlated with purine alkaloid response, namely the green module (908 genes), green–yellow module (267 genes), black (451 genes) and brown (1376 genes). Interestingly, the genes in the green module and green–yellow module are more strongly associated with caffeine and theobromine, especially in the green module. The genes for black and brown are closely associated with theacrine and total purine alkaloids, especially in the brown module. The DEGs enriched in the purine alkaloid pathway were screened out, the results showed that the DEGs in the green module were downregulated in JC, and there were mainly six genes, namely, *ADSL*, *RRM1*, *PK*, *5’-NT*, *UAZ* and *RRM1*. *UAZ* was differentially expressed at the three flowering periods. The genes in the brown module were all upregulated in JC, and there were five primary genes, namely *ADK*, *ADSL*, *TTHL*, *APRT*, *ADS*, *ADSL*, *APRT* and *ADSL*, which were all differentially expressed genes at the three flowering periods. The DEGs screened by WGCNA were consistent with previous studies. These genes could be used as the key regulatory genes for the formation of theacrine in tea blossoms, providing a specific theoretical basis and guidance for subsequent studies.

## 4. Materials and Methods

### 4.1. Plant Materials

The flowers of Kucha variety (Jiaocheng Kucha, JC) and conventional variety (Fuding Daibaicha, FD) with highly contrasting theacrine contents were used in this study. Three different flowering stages were collected. There were FDB and JCB (at flower bud stage), FDW and JCW (at whitening stage), FDF and JCF (at full opening stage). The Kucha variety were collected from Jiaocheng, Ningde, Fujian Province, and the conventional variety came from the same location. Flowers of each period were harvested in November 2020. The materials were stored at −80 °C for RNA isolation and purine alkaloids analysis.

### 4.2. Determination of Purine Alkaloids

Purine alkaloids were extracted with 30 mL of methanol, ultrasonicated for 30 min, and centrifuged at 10,000× *g* for 5 min. Then, taking supernatant through 0.22 μm membranethe to UPLC–MS/MS analysis, using a Nexera X2 LC-30A HPLC system (Shimadzu, Kyoto, Japan) and a tandem Sciex 4500 Q-Trap mass spectrometer (Sciex, Massachusetts, USA), UPLC–MS/MS was performed with a column temperature of 40 °C, the wavelength of 231 nm, injection volume of 5 μL and flow rate of 0.3 mL/min. A C18 column (2.6 µm, 2.1 × 100 mm) (Philomen, Guangzhou, China) with solvent A (0.1% formic acid) and solvent B (acetonitrile) was used as the mobile phase. The gradient programs were as follows: 0–0.2 min, linear gradient from 0 to 10% B; 0.2–2.5 min, linear gradient from 10 to 90% B; 2.5–4 min, 90% B; and 4–4.2 min, linear gradient from 90 to 10% B. Mass spectrometry conditions: electrospray source (ESI), positive ion mode, curtain gas 30 psi, electrospray voltage 4500 V, auxiliary gas (N_2_) temperature 550 °C, spray gas (N_2_) pressure 55 psi, auxiliary heating gas (N_2_) pressure 55 psi. All samples were repeated three times. MS parameters for the three purine alkaloids are given in Table 2.

### 4.3. Acquisition and Analysis of Transcriptome Sequencing

Total RNA was extracted using Plant RNA Purification Reagent (Invitrogen, State of California, USA), and RNA quality was assessed on an Agilent 2100 Bioanalyzer (Agilent Technologies, State of California, USA) and ND-2000 (Thermo, Waltham, USA) [30]. The transcriptome library was constructed according to the Illumina TruSeqT mRNA Sample Preparation Kit method, and the libraries were sequenced on the Illumina NovaSeq 6000 sequencing platform by Shanghai Meji Biopharma Technology Co., Ltd. SeqPrep (https://github.com/jstjohn/SeqPrep 25 March 2021) and Sickle (https://github.com/najoshi/sickle 25 March 2021) were used for quality control of the raw data and to obtain high-quality data. Using TopHat 2.1.1 software quality control data compared to the ‘Tie Guanyin’ genome [31], the difference between this study and the original annotation was obtained by using Cufflinks software, and the gene expression level was calculated by using RESM software based on TPM.

### 4.4. Differentially Expressed Genes and Enrichment Analysis

To screen out differentially expressed genes (DEGs), the EdgeR package (http://www.bioconductor.org/packages/2.12/bioc/html/edgeR.html 15 April 2021) was used [32]. We determined genes as |log2FoldChange| > 1, *p* value < 0.05. Foldchange represents the ratio of expression levels between the two sample groups. Goatools and KOBAS software were used to perform GO and KEGG functional enrichment analysis of differentially expressed genes, and when the corrected *p* value < 0.05, the GO function and KEGG pathway function were considered significantly enriched [33]. TBtools software was used to make a heat map for visualization of gene expression [34].

### 4.5. Coexpression Module Construction and Analysis

Background correction and standardization of gene expression data using RSEM software to filter genes with low expression and low coefficient of variation [35]. Construction of gene co-expression network using R software (R version 3.4.4) and WGCNA (Rversion 1.6.6) packages [36]. Gene modules highly related to metabolites were identified based on filtered data (average expression level 1, coefficient of varia-tion 0.1). Filtered abundance of 17,718 genes and three metabolites was used to build a co-expression network by calculating Pearson’s correlation.

### 4.6. Verification of Transcriptome Data

Primer3 plus online website (http://www.bioinformatics.nl/cgi-bin/primer3plus/Primer3plus. Cgi 25 June 2021) was used to design primers (Table S2), and cDNA was synthesized from RNA using the All-Gold EasyScript One-Step GDNA Removal and cDNA Synthesis Supermix kit (Quansi Gold Biotechnology Co., Ltd., Beijing, China). Referring to the method of Wang et al. [37], tea tree *GAPDH* (registration number *GE651107*) was selected as the internal reference gene [38]. qRT-PCR analysis was performed on a CFX96 Touch fluorescent quantitative PCR instrument (Bole Life Medical Products Co. Ltd., Shanghai, China) according to the instructions of the TransStart^®^Tip Green qPCR Supermix kit (Quansi Gold Biotechnology Co., Ltd., Beijing, China). Reaction procedures: 94 °C for 30 s; 94 °C for 5 s; 60 °C for 30 s; 40 cycles and three biological replicates. The 2^−ΔΔCT^ algorithm was used to calculate the relative gene expression level [39]. The histogram was made in prism 8.0.

### 4.7. Statistical Analysis

The data results were expressed as Mean ± SD, and the histogram was drawn by Prism 8.0. Analysis of variance and significant difference analysis by SPSS 19.0.

## 5. Conclusions

A transcriptomic approach was used to reveal the mechanism of theacrine formation of Jiaocheng kucha’s flower. Quantitation of purine alkaloid during all flowering stages revealed its changing tend. To sum up, the theacrine in the young bud stage was significantly higher than the other two stages. Transcriptome analysis echoed that DEGs on the purine alkaloid pathway was mainly related to xanthine synthesis, which was upregulated among bitter tea varieties, while it was downregulated on the caffeine anabolic pathway. A total of thirteen modules were identified through weighted correlation network analysis (WGCNA) method, and most transcriptome was correlated to the purine alkaloid. Furthermore, two key gene modules, green module and brown module, were screened for high correlation with purine alkaloid, among which *ADSL* (*CsTGY03G0002327), ADSL* (*CsTGY09G0001824) and UAZ*(*CsTGY06G0002694*) could be key genes attribute to theacrine synthesis of bitter tea flowers.

## Figures and Tables

**Table 1:** Data assembly for transcriptome sequencing.

Sample ID	Raw Reads (Gb)	Clean Reads (Gb)	Clean Bases (Gb)	Q20 (%)	Q30 (%)	GC Content (%)	Uniquely Mapped (%)
FDB-1	53.31	52.96	7.61	98.51	95.27	45.14	85.54
FDB-2	54.64	54.30	7.80	98.59	95.52	44.88	85.29
FDB-3	51.87	51.54	7.42	98.52	95.32	45.07	84.83
Average	53.27	52.93	7.61	98.54	95.37	45.03	85.22
FDW-1	59.03	58.66	8.49	98.5	95.26	45.3	86.27
FDW-2	61.01	60.64	8.78	98.54	95.39	45.52	86.44
FDW-3	58.60	58.22	8.37	98.53	95.39	45.75	85.76
Average	59.55	59.17	8.55	98.52	95.35	45.52	86.16
FDF-1	55.09	54.79	7.91	98.53	95.37	45.34	85.15
FDF-2	56.64	56.31	8.09	98.52	95.35	45.16	84.48
FDF-3	54.08	53.74	7.72	98.47	95.21	45.57	83.30
Average	55.27	54.95	7.91	98.51	95.31	45.36	84.31
JCB-1	53.94	53.50	7.63	98.61	95.61	44.53	83.06
JCB-2	57.28	56.86	8.15	98.6	95.58	44.42	83.23
JCB-3	56.09	55.65	7.96	98.6	95.57	44.54	83.12
Average	55.77	55.34	7.91	98.60	95.59	44.50	83.14
JCW-1	54.16	53.77	7.69	98.63	95.65	44.75	83.48
JCW-2	49.22	48.77	6.93	98.51	95.4	44.34	83.51
JCW-3	62.65	62.15	8.83	98.54	95.43	44.97	83.97
Average	55.34	54.90	7.82	98.56	95.49	44.69	83.65
JCF-1	57.85	57.46	8.33	98.49	95.3	45.78	79.05
JCF-2	52.88	52.50	7.55	98.48	95.27	45.48	80.13
JCF-3	57.21	56.81	8.16	98.45	95.19	45.93	78.60
Average	55.98	55.59	8.01	98.47	95.25	45.73	79.26

**Table 2:** UPLC-MS/MS parameters of purine alkaloids.

Purine Alkaloid	Retention Time (min)	Precurser Ion (*m/z*)	Product Ion *(m/z*)	Decluster Voltage (e/V)	Collision Energy (e/V)
Caffeine (CAF)	2.55	195.2	138.1 a/110.2/83.0	80.0	25.0/31.0/35.0
Theacrine (TCR)	2.47	225.1	168.1 a/153.2/210.1	80.0	27.2/34.0/27.3
Theobromine (TB)	1.85	181.0	107.9 a /122.1/67.10	91.3	30.0/35.0/43.0

Note: a represents ion pair selected for MRM quantity.

## Supplementary Materials

The following are available online at https://www.mdpi.com/article/10.3390/ijms222413394/s1.

## Abbreviations

JC: Jiaocheng kucha flowers; FD, Fuding Dabaicha flowers; UPLC-MS/MS, ultra-high performance liquid chromatography mass spectrometer; CAF, Caffeine; TB, Theobromine; TCR, Theacrine; TC, the total content of purine alkaloids; DEGs, differentially expressed genes; WGCNA, weighted correlation network analysis.
